# The Intersection of Autoimmunity and Sleep: Anti-IgLON5 as the Key Player in a Case Series

**DOI:** 10.7759/cureus.113719

**Published:** 2026-07-31

**Authors:** Benjamin Earnest Williams, Aditya Vijaykrishnan Nair, VigneshKumar Kathiresan, Ajith Sivadasan, Balamugesh Thangakunam, Barney Isaac, Devasahayam Jesudas Christopher, Vivek Mathew, Sanjith Aaron, John AJ Prakash

**Affiliations:** 1 Pulmonary Medicine, Christian Medical College and Hospital Vellore, Vellore, IND; 2 Neurological Sciences, Christian Medical College and Hospital Vellore, Vellore, IND; 3 Microbiology, Christian Medical College and Hospital Vellore, Vellore, IND

**Keywords:** anti-iglon5, autoimmune sleep disorder, iglon5, polysomnography, sleep disorders

## Abstract

Anti-IgLON5 disease is a recently identified autoimmune condition characterized by unique sleep disturbances and a broad spectrum of neurological symptoms. Alongside prominent manifestations such as bulbar dysfunction, gait abnormalities, movement disorders, and cognitive decline, affected individuals often experience severe and characteristic sleep disorders. These include parasomnias during both rapid eye movement (REM) and non-REM (NREM) sleep, stridor, and obstructive sleep apnea (OSA). The presence of antibodies targeting IgLON5, a cell adhesion protein with a still unclear role, is a defining feature of the disease. Due to the limited number of reported cases and studies, anti-IgLON5 disease remains an important and emerging area of interest in sleep medicine research.

This case series of seven patients highlights the importance of evaluating sleep-related symptoms not only in the context of sleep disorders but also through a comprehensive neurological history and examination. Such an approach is essential for identifying potential underlying conditions such as bulbar dysfunction, movement disorders, and cognitive impairment, which may indicate the rare autoimmune condition known as anti-IgLON5 disease.

## Introduction

Between 2010 and 2014, eight patients presenting with varied clinical features, including gait instability, abnormal movements, and bulbar dysfunction (such as dysphagia, dysarthria, and central hypoventilation), along with a distinctive sleep disorder involving non-rapid eye movement (NREM) and rapid eye movement (REM) parasomnias, obstructive sleep apnea (OSA), and stridor, were diagnosed with anti-IgLON5 disease following the identification of IgLON5 antibodies [1]. This discovery has since brought significant attention to the condition among sleep physicians and neurologists. However, awareness of this new entity is sparse, and reports from Asia remain limited, with fewer than 30 cases reported in the literature so far.

We report a case series of seven patients with anti-IgLON5 disease. This case series draws attention to the predominant role of sleep dysfunction in the clinical spectrum of the disease. The study underscores the diagnostic importance of polysomnography (PSG) in identifying hallmark sleep abnormalities and emphasizes the need for early diagnosis and prompt immunotherapy. These findings advocate for a collaborative, multidisciplinary approach integrating sleep specialists and neurologists to enhance diagnostic precision and improve patient outcomes.

## Materials and methods

Study design and setting

This retrospective case series was conducted at the Department of Pulmonary Medicine (Sleep Disorders Centre) and the Department of Neurology, Christian Medical College and Hospital, Vellore, Tamil Nadu, India, a quaternary care teaching hospital. Patients evaluated between January 2022 and December 2025 were screened for inclusion.

Study participants

Patients were eligible for inclusion if they fulfilled all of the following criteria: clinical suspicion of anti-IgLON5 disease based on neurological and sleep-related manifestations; positive serum anti-IgLON5 antibody serology confirming the diagnosis; and availability of complete clinical records and overnight attended PSG or a level 3 sleep study performed at our sleep laboratory. Patients with negative anti-IgLON5 antibody serology were excluded from the analysis. A total of seven patients fulfilled the eligibility criteria and were included in the study.

Clinical assessment

Clinical data were retrieved from the institutional electronic medical records and included demographic characteristics, presenting symptoms, neurological manifestations, sleep-related complaints, cognitive symptoms, bulbar dysfunction, movement disorders, autonomic symptoms, neurological examination findings, laboratory investigations, neuroimaging findings, electrophysiological studies where available, treatment received, and clinical outcomes.

All patients underwent diagnostic evaluation by neurologists, including serum anti-IgLON5 antibody testing. Additional investigations, including magnetic resonance imaging (MRI) of the brain, cerebrospinal fluid (CSF) analysis, electroencephalography (EEG), nerve conduction studies/electromyography (NCS/EMG), and other investigations, were performed as clinically indicated to exclude alternative diagnoses.

Sleep assessment

Sleep-related symptoms were evaluated using clinical history and the Epworth Sleepiness Scale (ESS), a validated eight-item questionnaire that assesses the subject's likelihood of dozing during common daytime situations, with total scores ranging from 0 to 24. Higher scores indicate greater subjective daytime sleepiness. ESS scores were interpreted as follows: 0-10, normal daytime sleepiness; 11-12, mild excessive daytime sleepiness (EDS); 13-15, moderate EDS; and 16-24, severe EDS. The ESS was administered prior to PSG during the initial sleep evaluation [2].

All patients underwent overnight attended level I PSG at the institutional sleep laboratory. The studies included EEG, electrooculography, chin and bilateral anterior tibialis EMG, electrocardiography, airflow monitoring using nasal pressure transducer and oronasal thermal sensor, thoracoabdominal respiratory effort belts, pulse oximetry, body position monitoring, snore microphone, and synchronized video recording. Level 3 sleep study for airflow monitoring, respiratory effort, body position, and pulse oximetry recording was also accepted if the patient did not agree to level 1 PSG.

Polysomnographic recordings were manually scored according to the American Academy of Sleep Medicine (AASM) Manual for the Scoring of Sleep and Associated Events, Version 3 [3]. Sleep architecture, sleep efficiency, respiratory events, oxygenation parameters, limb movements, parasomnias, and other abnormal sleep-related motor behaviors were reviewed.

Study outcomes

The primary outcome was to describe the clinical and polysomnographic characteristics of patients with serologically confirmed anti-IgLON5 disease. The secondary outcome was to describe the spectrum of sleep-disordered breathing in anti-IgLON5 disease.

Ethical considerations

The study was conducted in accordance with the principles of the Declaration of Helsinki. Institutional Ethics Committee approval was obtained prior to data collection, and patient confidentiality was maintained throughout the study.

## Results

Case descriptions

Clinical Findings

Six male patients aged between 42 and 73 years and one female aged 64 years (mean age 58 years) presented with symptoms of sleep-disordered breathing and bulbar symptoms such as dysphagia and dysphonia. The initial symptoms in four patients were sleep-related EDS and dream enactment. In two others, the initial presentation was with bulbar symptoms and choreiform movements in one patient. Detailed demographic and clinical information is provided in Table 1.

**Table 1:** Demographic and clinical information EDS: Excessive daytime sleepiness; OSA: Obstructive sleep apnea; RBD: REM behavior disorder; REM: Rapid eye movement; NREM: Non-rapid eye movement; PLMS: Periodic limb movement of sleep; IVIG: Intravenous immunoglobulin.

	Case 1	Case 2	Case 3	Case 4	Case 5	Case 6	Case 7
Age at diagnosis	57	58	64	42	73	51	63
Gender	M	M	F	M	M	M	M
Time from onset to diagnosis (months)	36	7	1	10	5	16	24
Symptoms at onset	Loud snoring	EDS	Dysphagia	EDS	Dysphonia	Dream enactments	Chorea
Symptoms at diagnosis	Dysphagia, EDS	EDS, disturbed sleep at night, slurring of speech	Dysphagia, left eye ptosis, EDS, snoring, dream enactment	Sleep disturbances, limb movements during sleep, insomnia, gait and posture abnormality	Dysphonia, dysphagia, gait abnormality, EDS	EDS, dream enactments, disturbed sleep with recurrent arousals	Slowness of movements, chorea, EDS
Bulbar symptoms	Dysphagia, dysphonia, sialorrhea	Slurred speech	Dysphagia	No	Dysphagia, dysphonia	No	No
Cognitive impairment	No	Yes	No	Yes	No	No	No
Movement disorder	No	No	No	Yes, drooped posture	Yes	No	Yes, slowness of movements
Sleep disorder	OSA, EDS (1 year before bulbar symptoms)	OSA (predominant complaint)	OSA, RBD	OSA, NREM parasomnias-sleep talking	OSA	Mild OSA, RBD, PLMS	OSA, EDS
Anti-IGLON5							
Serum	Positive	Positive	Positive	Positive	Positive	Positive	Positive
CSF	Positive	N/A	N/A	N/A	N/A	N/A	N/A
Treatment	Inj methyl prednisolone, Inj rituximab	IVIG, Inj rituximab	IVIG, Inj rituximab	Inj methyl prednisolone, Inj rituximab	Inj methyl prednisolone, Inj rituximab	Inj methyl prednisolone	Lost follow-up
Last follow-up (months)	9	15	3	6	2	N/A	N/A
Response to immunotherapy	Bulbar symptoms and sleep improved	Bulbar symptoms and sleep improved	Improvement in bulbar symptoms, partial improvement in sleep/RBD	Improvement in daytime sleepiness, alertness, hallucinations, and parasomnias persisted. Stereotypic neck flexion movements persisted.	Drowsiness persisted	Lost follow-up	Lost follow-up

The time from symptom onset to diagnosis of anti-IgLON5 disease ranged from one month to three years, with a median duration of 14 months. All patients reported EDS, with a mean ESS score of 11.2. Cases 2 and 6 experienced near-miss accidents while driving due to their sleepiness. Bulbar symptoms, including dysphagia, dysphonia, and sialorrhea, were present in four patients (cases 1, 2, 3, and 5). Patient 1 had a history of loud, stridor-like snoring, which showed improvement with BPAP subsequently. OSA was confirmed in all patients through overnight PSG or limited-channel sleep studies. Three patients (cases 3, 4, and 6) exhibited REM sleep behavior disorder (dream enactments), and patient 4 experienced sleep talking, hallucinations, and stereotyped neck flexion movements during sleep. Three patients (cases 4, 5, and 7) had movement disorders with slowness of movements and chorea. The overlap of symptom complexes and sleep disorder subtypes in our patients is shown in Tables 2, 3.

**Table 2:** Symptom complex

Patient	Sleep disorder	Bulbar symptoms	Cognitive impairment	Movement disorders
1	Yes	Yes	No	No
2	Yes	Yes	Yes	No
3	Yes	Yes	No	No
4	Yes	No	Yes	Yes
5	Yes	Yes	No	Yes
6	Yes	No	No	No
7	Yes	No	No	Yes

**Table 3:** Sleep disorder subtypes OSA: Obstructive sleep apnea; REM: Rapid eye movement; PLMS: Periodic limb movement of sleep.

Patient	OSA	Parasomnia	PLMS	REM Stage
1	Yes	No	No	Decreased
2	Yes	No	No	Absent
3	Yes	Yes	Yes	Decreased
4	Yes	Yes	Yes	Normal
5	Yes	No	No	Absent
6	Yes	Yes	Yes	Increased
7	Yes	No	No	Decreased

Investigations

All seven patients tested positive for IgLON5 antibodies in their serum, while one patient also had the antibodies detected in CSF. Antibody to the IgLON5 gene was detected by cell-based immunofluorescence assay using a commercially available kit (Euroimmun, Germany). Brain MRI scans were normal in all cases. NCS, including repetitive nerve stimulation (RNS), showed no abnormalities. Additionally, tests for anti-acetylcholine receptor antibodies and anti-MuSK antibodies - used to evaluate for bulbar-onset myasthenia gravis - were negative in patients who presented with bulbar symptoms.

Sleep Study 

Details of the sleep study findings are summarized in Table 4.

**Table 4:** Sleep symptoms and sleep study findings AHI: Apnea-hypopnea index; DI: Desaturation index; EDS: Excessive daytime sleepiness; ESS: Epworth sleepiness scale; LSAT: Lowest saturation; OSA: Obstructive sleep apnea; RBD: REM behavior disorder; REM: Rapid eye movement; NREM: Non-rapid eye movement; PLMS: Periodic limb movement of sleep; PLMI: Periodic limb movement index; WASO: Wake after sleep onset.

	Case 1	Case 2	Case 3	Case 4	Case 5	Case 6	Case 7
Sleep apnea	OSA	OSA	OSA	OSA	OSA	OSA	OSA
Parasomnia	No	No	RBD - Dream enactments	RBD - Dream enactments, sleep talking	No	RBD - Dream enactments	No
Excessive daytime sleepiness	Yes	Yes	Yes	Yes	Yes	Yes	Yes
ESS	5	12	11	12	11	16	11
Sleep efficiency (%)	92%	13.7%	54.80%	-	20.3%	98.80%	29%
Sleep latency (min)	2	28.1	11.5	-	32	1.5	60
Sleep architecture	Decreased REM	Absent REM	Altered, increased N1, decreased N3, and REM	Altered	Altered, absent REM	REM increased	Decreased REM
REM latency (mins)	287	-	133.5	-	-	266.5	259
N1 (%)	26.5	72.1	61.1	-	79.1	1.7	26.4
N2 (%)	57.4	21.7	36.8	-	18.3	46.9	46.6
N3 (%)	15.3	6.2	1.1	-	2.6	12.4	25.5
REM (%)	0.9	0	1.1	-	0	39	2.4
WASO (mins)	33	376	1.4	-	194	3.7	195
AHI (events/hour)	12.5	12 and 6	65.6	58.8	6.3	7	32.3
DI (events/hour)	15	44.5	55	60.7	4.2	7	32.9
LSAT (%)	85	87	69	58	93	87	89
PCO_2 _(mmHg)	35	53	47	56	-	39	35
PLMI (events/hour)	18.5	204.7	16.7	5.2	34.4	74	4.6
Arousal index (per hour)	5.8	35.3	40.3	66.8	9.4	0	13.3
CPAP/BPAP trial	BPAP	CPAP/BPAP	BPAP	BPAP	Nil	Nil	Lost follow-up
BPAP pressures	16 and 6	18 and 10	14 and 10	12 and 6	Nil	Nil	-
Compliance	80%	97%	Good	Poor	Nil	Nil	-
Follow-up	Significant improvement	Significant improvement	Partial improvement	Partial improvement	Drowsiness persisted	Lost to follow-up	Lost to follow-up

Two patients demonstrated good sleep efficiency, while four had poor efficiency. All patients showed disrupted sleep architecture, with reduced REM sleep in three cases and increased REM in one case. There were frequent arousals in three patients with a significant arousal index (patients 2, 3, and 4). Sleep latency was shortened in three patients. Patient 6 exhibited marked EDS and was evaluated for narcolepsy. The Multiple Sleep Latency Test (MSLT) revealed a sleep latency of less than 8 minutes in all three naps, with a mean sleep latency of 0.67 minutes and the presence of three sleep-onset REM periods (SOREMPs).

**Figure 1:**
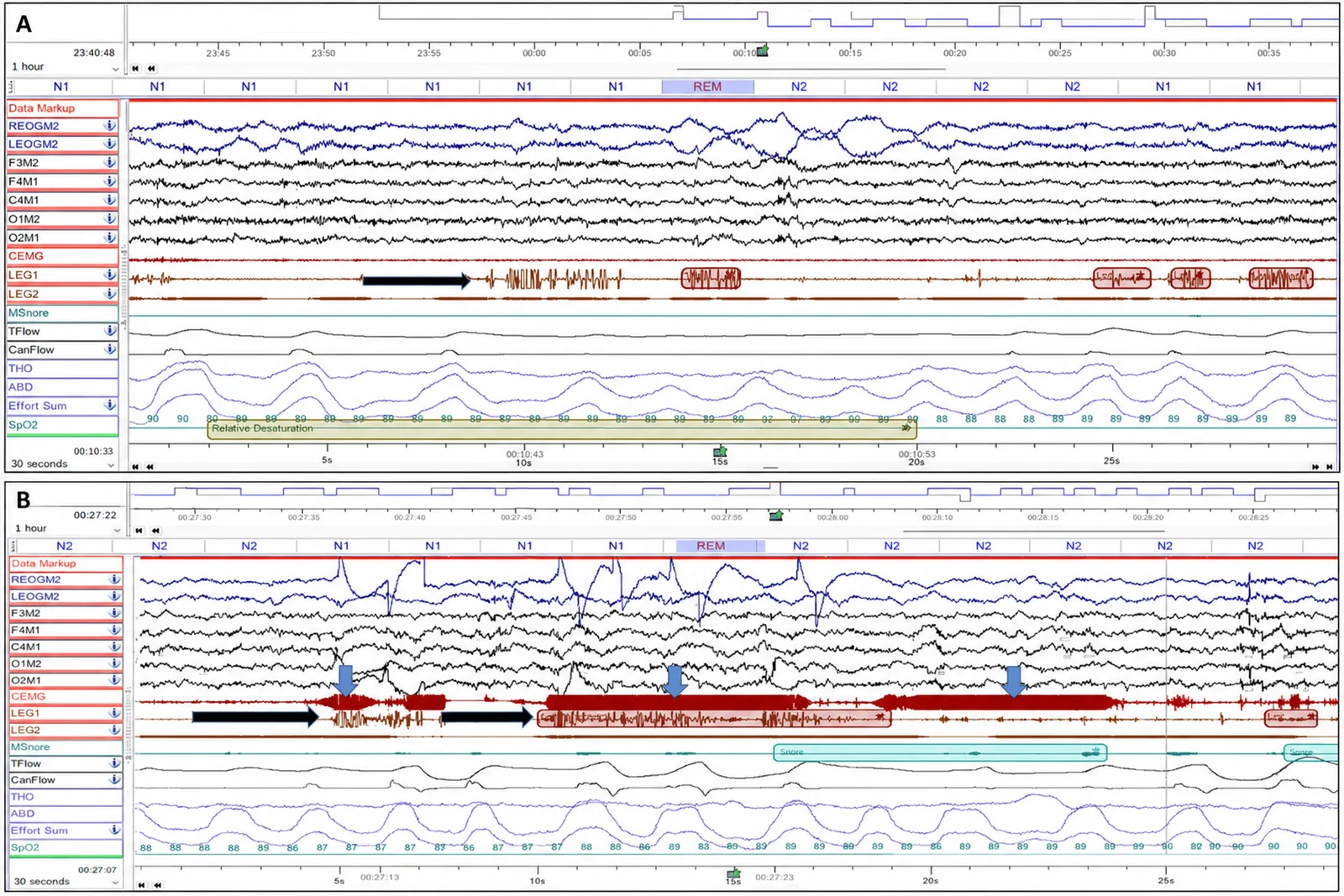
Representative polysomnographic epochs demonstrating REM sleep without atonia (RWA) in Patient 3 with anti-IgLON5 disease (A) Thirty-second polysomnographic epoch scored as REM sleep, demonstrating preserved physiological chin electromyographic atonia despite the presence of phasic limb electromyographic activity (black arrow), consistent with isolated REM-related limb movements. (B) Thirty-second REM sleep epoch demonstrating RWA, characterized by sustained tonic chin EMG activity (blue arrows) together with concurrent sustained leg EMG activity (black arrows), indicating loss of normal REM muscle atonia. REM: Rapid eye movement; RWA: REM sleep without atonia; EMG: Electromyography.

Across all patients, the average apnea-hypopnea index (AHI) and desaturation index (DI) were 30.4 and 31.3 events per hour, respectively. The mean lowest oxygen saturation recorded was 81%, and hypoventilation was noted in three patients (patients 2, 3, and 4). Periodic limb movement disorder (PLMD) was identified in five patients, each having a PLMS index greater than 15, as defined by the International Classification of Sleep Disorders, Third Edition (ICSD-3) criteria. A PSG image of EPOCHs scored as REM stage sleep with loss of REM atonia exhibited by patient 3 with dream enactment is shown in Figure 1.

Treatment and Follow-Up

Following the confirmation of anti-IgLON5 disease through serum antibody testing, six patients received immunotherapy, including methylprednisolone injections and rituximab. One patient was lost to follow-up. Four patients were started on positive airway pressure (PAP) therapy (continuous positive airway pressure (CPAP) or bilevel positive airway pressure (BPAP)) after an initial trial in the lab. Among those, two patients who adhered to the therapy showed notable improvement in sleep-related symptoms. Partial improvement was observed in three out of six patients, and one patient was lost to follow-up. Bulbar symptoms showed improvement in all four patients who received immunotherapy. None of the patients had progressed to respiratory failure on a mean follow-up of seven months.

## Discussion

Anti-IgLON5 disease is a rare autoimmune encephalopathy first identified by Sabater et al. in 2014, characterized by autoantibodies targeting the neuronal cell adhesion protein IgLON5 [1]. One of its hallmark features is the presence of severe and at times life-threatening sleep disturbances that frequently dominate the clinical presentation. Sleep-related dysfunction plays a central role in the disease’s manifestation. As highlighted by Gelpi et al. (2016), over 80% of patients experience sleep disturbances, often appearing before or masking other neurological symptoms [4]. IgLON5 antibodies cause irreversible reduction of IgLON5 protein on neuronal surfaces, causing loss of nerve cells along with the buildup of an abnormal form of a protein called tau. This protein, which normally helps support brain cells, becomes damaged and clumps together, thus disrupting cytoskeletal integrity and possibly contributing to sleep dysfunction. These clumps are mostly found in areas of the brain that control vital functions, such as the hypothalamus and the brainstem. It follows a rostrocaudal gradient, extending through the hippocampus, periaqueductal gray matter, medulla oblongata, and upper cervical spinal cord. Graus et al. (2025), in a 10-year collection of 98 cases, showed that the sleep symptoms were reported by 84% of patients or their bed partners; 63% had sleep breathing problems (e.g., apneas, snoring, and stridor), 50% exhibited sleep-related vocalizations and abnormal behaviors, 54% reported insomnia, and 47% had EDS [5]. In our cohort of patients with confirmed IgLON5 disease, all individuals exhibited sleep-related symptoms, which led four of them to seek medical evaluation.

Key sleep abnormalities include sleep-disordered breathing. Central and OSA are the most notable issues, often accompanied by stridor and hypoventilation during sleep (Gaig et al., 2017) [6]. This led to marked nocturnal oxygen desaturation and EDS. All patients in our series were diagnosed with OSA and daytime sleepiness. REM and NREM parasomnias are frequently observed as parasomnias and motor activity, with symptoms such as dream enactment, complex movements, and vocal outbursts, resembling REM sleep behavior disorder (RBD) (Honorat et al., 2017) [7]. Three of our patients exhibited RBD in the form of dream enactments during sleep. Sleep studies commonly show reduced sleep efficiency, fragmented sleep cycles, non-REM and REM parasomnia with finalistic movements, and sleep-disordered breathing (stridor, respiratory failure, and OSA). There may also be changes in sleep spindle activity and slow-wave sleep, indicating altered sleep regulation [8]. Graus et al. (2025), in their 98 cases, found that there were characteristic findings in 68% of patients who underwent video polysomnography (v-PSG), including abnormal initiation of sleep with undifferentiated NREM stages, REM sleep behavior disorder (RBD), episodes of stridor, and OSA [5]. Narcolepsy with cataplexy could be a secondary manifestation of anti-IgLON5 disease, caused by an autoimmune attack on the hypothalamus by anti-IgLON5 disease antibodies [9]. Patient 6 in our series was also diagnosed with narcolepsy without cataplexy clinically and by MSLT.

Management of anti-IgLON5 disease involves both immunomodulatory therapies and interventions aimed at controlling sleep symptoms. Treatment typically involves high-dose corticosteroids, intravenous immunoglobulin (IVIG), plasmapheresis (PLEX), or rituximab. According to Gaig et al. (2017), approximately 40%-50% of patients report partial relief of sleep-related symptoms, especially those involving breathing, although long-term outcomes vary [6]. In a pooled cohort of 46 patients with anti-IgLON5 disease, immunotherapy produced a response in 43% overall and 33% at the last follow-up, with higher response rates for combination therapy (67%) and second-line therapy (54%). Response rates by the agent were 34% for steroids, 43% for IVIG, 46% for PLEX, 100% for azathioprine, and 75% for mycophenolate. Favorable responses correlated with cognitive impairment, non-classical phenotypes, HLA (human leukocyte antigen)-DQB105:01 (without DRB110:01), and CSF inflammation [10]. In our series, four of the seven patients demonstrated improvement in bulbar and sleep-related symptoms during follow-up. One patient continued to experience daytime drowsiness, while two were lost to follow-up.

PAP therapy (CPAP or BPAP) is commonly employed for managing central and OSA. Symptom improvement has been noted in several cases, although motor symptoms may affect treatment adherence. PAP therapy, while beneficial, may be limited by bulbar dysfunction, poor tolerance, or progressive respiratory failure, necessitating individualized ventilatory strategies and careful follow-up [11]. Clonazepam and melatonin are used to treat parasomnias and RBD-like behaviors.

Although immunotherapy may lead to clinical improvement, its long-term effectiveness is often constrained by the disease's dual pathology, comprising autoimmune processes coupled with neurodegeneration (Gelpi et al., 2016) [4]. Early treatment appears to be associated with more favorable outcomes, although definitive evidence remains limited. Mortality in anti-IgLON5 disease exceeds that of other autoimmune encephalitis syndromes, particularly in patients with respiratory involvement [1]. Reported causes of death most commonly include laryngeal spasm, respiratory failure, central hypoventilation, aspiration pneumonia, and cardiac complications [6,12-15], necessitating heightened clinical vigilance in this population.

This study has several limitations. First, its retrospective design is subject to incomplete clinical documentation and limits the ability to establish temporal or causal relationships between sleep abnormalities and disease progression. Second, although our cohort represents one of the largest reported single-center series from Asia, the small sample size reflects the rarity of anti-IgLON5 disease and limits the generalizability of our findings and the ability to perform meaningful statistical analyses. Third, not all patients underwent uniform longitudinal follow-up, and two patients were lost to follow-up, limiting the assessment of long-term clinical outcomes and responses to immunotherapy and PAP therapy. Finally, as a tertiary referral-center study, referral bias may have resulted in an overrepresentation of patients with prominent sleep manifestations. Nevertheless, this series provides valuable clinical and polysomnographic insights into this uncommon disorder and highlights the importance of recognizing characteristic sleep features that may facilitate earlier diagnosis.

Further research is needed to clarify the mechanisms by which IgLON5 antibodies disrupt sleep. Due to the lack of large studies that clearly indicate the advantage of one drug over another or determine the optimal time to initiate treatment, further research involving larger patient cohorts is required.

## Conclusions

Sleep disturbances are a key feature of the clinical presentation in anti-IgLON5 disease, commonly appearing as sleep-disordered breathing, parasomnias, and significant alterations in sleep structure. While immunotherapy and supportive care can offer some improvement, managing the condition is challenging due to its underlying neurodegenerative nature. Comprehensive multidisciplinary care and coordination between neurologists, sleep specialists, and pulmonologists are critical for tailoring treatment strategies and managing disease progression effectively. Continued research is needed to advance more precise and effective therapeutic options.
